# Efficacy of *Origanum syriacum* Essential Oil against the Mosquito Vector *Culex quinquefasciatus* and the Gastrointestinal Parasite *Anisakis simplex*, with Insights on Acetylcholinesterase Inhibition

**DOI:** 10.3390/molecules24142563

**Published:** 2019-07-15

**Authors:** Víctor López, Roman Pavela, Carlota Gómez-Rincón, Francisco Les, Fabrizio Bartolucci, Veronica Galiffa, Riccardo Petrelli, Loredana Cappellacci, Filippo Maggi, Angelo Canale, Domenico Otranto, Stefania Sut, Stefano Dall’Acqua, Giovanni Benelli

**Affiliations:** 1 Department of Pharmacy, Faculty of Health Sciences, Universidad San Jorge, Villanueva de Gállego, 50830 Zaragoza, Spain; 2 Instituto Agroalimentario de Aragón-IA2 (CITA-Universidad de Zaragoza), 50013 Zaragoza, Spain; 3 Crop Research Institute, Drnovska 507, 110 00-119 99 Prague, Czech Republic; 4 School of Bioscience and Veterinary Medicine, University of Camerino, Centro Ricerche Floristiche dell’Appennino, San Colombo, 67021 Barisciano, Italy; 5 School of Pharmacy, University of Camerino, via Sant’Agostino 1, 62032 Camerino, Italy; 6 Department of Agriculture, Food and Environment, University of Pisa, via del Borghetto 80, 56124 Pisa, Italy; 7 Department of Veterinary Medicine, University of Bari Aldo Moro, 70100 Bari, Italy; 8 Department of Agronomy, Food, Natural Resources, Animals and Environment (DAFNAE), University of Padova, 35020 Legnaro, Italy; 9 Department of Pharmaceutical and Pharmacological Sciences, University of Padova, 35128 Padova, Italy

**Keywords:** anisakiasis, contact toxicity, enzyme inhibition, fumigation toxicity, larvicide, mosquito control, penetration assay

## Abstract

Developing effective and eco-friendly antiparasitic drugs and insecticides is an issue of high importance nowadays. In this study, we evaluated the anthelminthic and insecticidal potential of the leaf essential oil obtained from *Origanum syriacum* against the L3 larvae of the parasitic nematode *Anisakis simplex* and larvae and adults of the mosquito *Culex quinquefasciatus*. Tests on *A. simplex* were performed by standard larvicidal and penetration assays, while mosquito toxicity was assessed relying on larvicidal, tarsal contact, and fumigation tests. To shed light on the possible mode of action, we analyzed the oil impact as acetylcholinesterase (AChE) inhibitor. This oil was particularly active on L3 larvae of *A. simplex*, showing a LC_50_ of 0.087 and 0.067 mg mL^−1^ after 24 and 48 h treatment, respectively. *O. syriacum* essential oil was highly effective on both larvae and adults of *C. quinquefasciatus*, showing LC_50_ values of 32.4 mg L^−1^ and 28.1 µg cm^−2^, respectively. Its main constituent, carvacrol, achieved larvicidal LC_50(90)_ of 29.5 and 39.2 mg L^−1^, while contact toxicity assays on adults had an LC_50(90)_ of 25.5 and 35.8 µg cm^−2^, respectively. In fumigation assays, the LC_50_ was 12.1 µL L^−1^ after 1 h and decreased to 1.3 µL L^−1^ in 24 h of exposure. Similarly, the fumigation LC_50_ of carvacrol was 8.2 µL L^−1^ after 1 h of exposure, strongly decreasing to 0.8 µL L^−1^ after 24 h of exposure. These results support the folk usage of Lebanese oregano as an antiparasitic agent, providing new insights about its utilization for developing new effective and eco-friendly nematocidal and insecticidal products.

## 1. Introduction

The development of new drugs to fight parasitic infections such as anisakiasis is highly needed. Anisakiasis, the parasitic infection of the gastrointestinal tract caused by the members of the genus *Anisakis,* such as *A*. *simplex* Dujardin larvae, is a public health concern worldwide, particularly in Asian countries such as Japan, and in Mediterranean areas, such as Spain and Italy [1,2]. The high prevalence of this zoonoses, also named as anisakidosis, is caused by the consumption of raw or undercooked fish or seafood, which leads to the ingestion of larvae of the Anisakidae family belonging to the genera *Anisakis*, *Pseudoterranova*, or *Contracaecum* [3]. In many cases, these infections are resolved without pharmacological treatments because larvae die in human hosts; a major risk is represented by allergic reactions and anaphylaxis caused by larvae antigens. Allergic reactions can be diagnosed through clinical features and elevation of Immunoglobulin E, whereas many infections are misdiagnosed, in particular due to the lack of specificity of the clinical symptoms (i.e., abdominal pain, vomiting, and diarrhea) [4].

Furthermore, the timely and effective control of insect vectors represents a crucial challenge in medical and veterinary entomology [5,6,7]. Mosquitoes within the genera *Anopheles*, *Aedes*, and *Culex* include some of the most dangerous and worldwide spread insect species, acting as competent vectors of malaria, dengue, yellow fever, West Nile, chikungunya, and Zika virus [8,9]. Among *Culex* species of medical relevance, *Culex quinquefasciatus* Say attracts the attention of scientists worldwide being the main vector of filariasis, currently recognized as one of the most important neglected tropical diseases [10,11], while its competence as a Zika virus vector is still debated [12,13,14]. The management of this mosquito species is based on the employment of synthetic insecticides though this is challenged by the quick development of resistance in targeted populations [15]. Therefore, in agreement with the Integrated Vector Management (IVM) [16] and One Health criteria [17,18], the development of novel and environmentally sustainable ovicides, larvicides, and pupicides to be used in aquatic environments, as well as adulticides and repellents, is a major target for current entomological research [19,20,21,22].

Plant secondary metabolites represent an ancient and huge source of bioactive molecules of potential interest for developing new insecticides [23,24,25] and antiparasitic drugs [26,27,28,29,30,31]. The genus *Origanum* L. (Lamiaceae) comprises 43 species (51 taxa) worldwide [32,33,34,35,36,37,38], with its center of diversity in the Mediterranean area [32]. According to the classification proposed by Ietswaart [32], based on morphological characters, *Origanum* is classified into ten sections.

*Origanum syriacum* L., also known as Biblical-hyssop, Lebanese oregano, or Syrian oregano, is distributed in the eastern Mediterranean area, especially in Turkey, Cyprus, Syria, Lebanon, Israel, Jordan and Egypt. It is found on rocky soils from the sea level up to about 2000 m of altitude [32]. *O. syriacum* belongs to the section *Majorana*, and its high morphological variation led to the recognition of three varieties [32]: *O. syriacum* var. *syriacum*, var. *bevanii* (Holmes) Ietsw., and var. *sinaicum* (Boiss.) Ietsw. More recently, these three taxa were reconsidered as subspecies [39]: *O. syriacum* subsp. *syriacum*, subsp. *sinaicum* (Boiss.) Greuter and Burdet, and subsp. *bevanii* (Holmes) Greuter and Burdet.

*O. syriacum* is one of the most important herbal remedies used in the folk medicine of the Middle East, especially in Lebanon, Israel, Jordan, Syria, and Egypt. In Lebanon, the plant leaves (known under the vernacular names of “Zoubà” and “Za’atar”) are used under infusion to treat nervous conditions, Alzheimer’s disease, rheumatic pains, respiratory and gastrointestinal ailments, diabetes, hypertension and worms [40,41,42,43,44].

Like other representatives of the genus *Origanum*, Za’atar is a rich source of essential oil (up to 6% w/w), which is mainly obtained from the leaves. This oil is appreciated for its noteworthy antioxidant and antimicrobial properties that make it an ideal food preservative [45,46,47]. From a phytochemical standpoint, two main essential oil chemotypes are reported for *O. syriacum*, i.e., the carvacrol-type and the thymol-type, though intermediate forms are frequently possible [46,48,49,50,51]. These ‘cymyl’ chemotypes are formed through the activity of the γ-terpinene synthase that drives the cyclization of geranyl pyrophosphate (GPP) into the intermediates γ-terpinene, *p*-cymene, and related compounds [48]. Some authors reported that the thymol chemotype occurs mostly in wild populations of *O. syriacum*, whereas the carvacrol chemotype occurs in cultivated ones [50].

Overall, although important biological properties have been recognized for *O. syriacum* essential oil, namely antimicrobial and antioxidant activities, as well as the leaf folk use—mixed with Shanklish cheese—for antiparasitic purposes [42], its insecticidal and anthelminthic potentials have been poorly explored so far. To the best of our knowledge this oil was assayed against the mosquito vector *Culex pipiens* L. [52] and the stored grain pests *Sitophilus zeamais* Motschulsky [53], *Tribolium confusum* du Val [54], and *Ephestia kuehniella* Zell. [55]. Furthermore, its nematocidal effects against *Meloidogyne javanica* (Treub.) have been evaluated [56]. *O. syriacum* essential oil has been also reported as a potential bioherbicide [57].

Based on the above, we hypothesized that the essential oil from this plant species may be exploited further as a useful source of compounds with antiparasitic and insecticide activity. Therefore, boosting our research line focused on disclosing new essential oils and isolated compounds with promising effectiveness against parasites and vectors of public importance [31,58,59,60], herein we evaluated the activity of the essential oil obtained from the Lebanese *O. syriacum* against the parasitic nematode *A. simplex,* through larvicidal and penetration assays. Furthermore, this oil was assessed for its bioactivity on the larvae of the mosquito vector *C. quinquefasciatus*. The main constituent of the essential oil, i.e., carvacrol, was also tested in mosquito larvicidal assays. Furthermore, the efficacy of *O. syriacum* essential oil and carvacrol on *C. quinquefasciatus* was assessed by adult toxicity (i.e., via tarsal and fumigation tests). The essential oil chemical composition was fully provided, relying on GC-MS analyses. Lastly, we investigated whether one of the possible modes of action of the *O. syriacum* essential oil may be the inhibition of acetylcholinesterase (AChE), an enzyme ensuring the breakdown of acetylcholine, which acts as a neurotransmitter in both invertebrate species. Therefore, AChE inhibition assays testing increasing concentrations of this essential oil were carried out, comparing its performances with the highly effective AChE inhibitor, galantamine.

## 2. Results

### 2.1. Essential Oil Extraction and Chemical Analysis

As reported in our recent study [61], hydrodistillation of leaves from the Lebanese *O. syriacum* gave a high essential oil yield (4.3%). The essential oil chemical profile was mostly made up of oxygen-containing monoterpenes (85.8%), with carvacrol as the most abundant component (82.6%). Other noteworthy constituents were γ-terpinene (5.7%), *p*-cymene (3.7%), thymol (2.4%), α-terpinene (1.3%), myrcene (1.0%), and (*E*)-caryophyllene (0.9%).

### 2.2. Anthelmintic Activity against A. simplex

**Figure 1:**
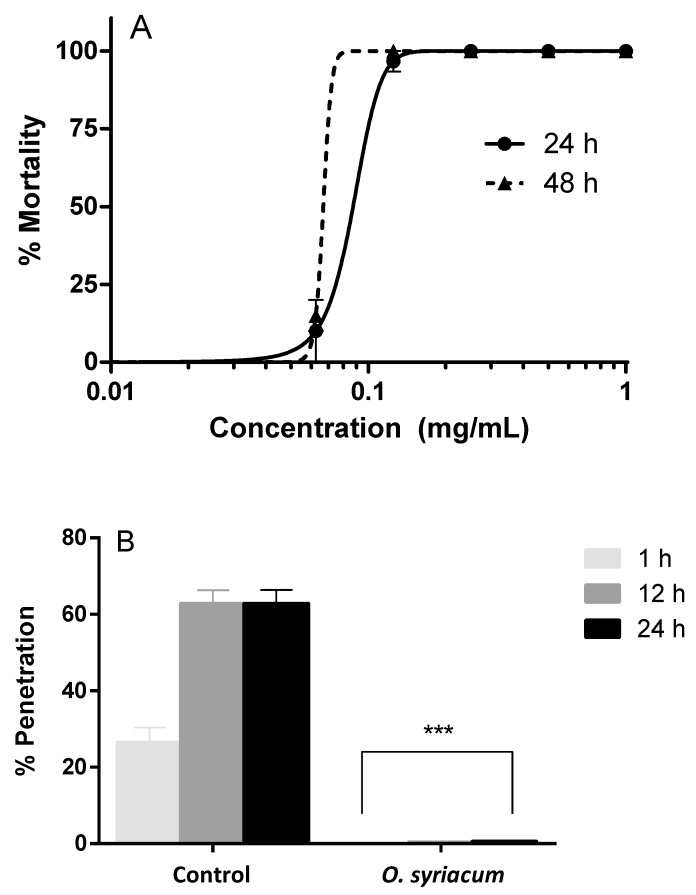
Anthelmintic activity of the *Origanum syriacum* essential oil: larvicidal activity against L3 larvae of *Anisakis simplex* after 24–48 h (**A**), larval penetration was fully inhibited after 1, 12, and 24 h of exposure to the oil, if compared to control wells (**B**). *** *p* < 0.001 versus control.

*O. syriacum* essential oil exerted larvicidal activity against *A. simplex* larvae, inducing causing 100% mortality at 0.125 mg mL^−1^ (Figure 1A). Median lethal concentration (LC_50_) values were 0.087 mg mL^−1^ after 24 h treatment and 0.067 mg mL^−1^ after 48 h. Penetration assay data showed that *A. simplex* larvae did not penetrate in the agar treated with *O. syriacum* essential oil at the LC_50_ concentration. Considering that in the control about 60% of *A. simplex* larvae were able to penetrate after 12 and 24 h from the start of the experiment, our results revealed a high reduction of the infective capacity of the parasites (Figure 1B).

### 2.3. Larvicidal, Tarsal, and Fumigation Activity on C. quinquefasciatus

Our insecticidal assays conducted on *C. quinquefasciatus* showed both larvicidal and adulticidal activity of the *O. syriacum* essential oil. Third instar larvae exposed to the essential oil showed LC_50(90)_ values of 32.4 and 40.1 mg L^−1^, respectively, while the oil major constituent carvacrol achieved LC_50(90)_ values of 29.5 and 39.2 mg L^−1^, respectively. The positive control α-cypermethrin had LC_50(90)_ values of 0.0008 and 0.0025 mg L^−1^, respectively (Table 1).

Furthermore, contact toxicity testing the essential oil and its main constituent, carvacrol, on adults assayed through the tarsal test led to LC_50(90)_ values of 28.1 and 46.9 µg cm^−2^ and 25.5 and 35.8 µg cm^−2^, respectively. The LC_50(90)_ values obtained testing α-cypermethrin were 1.22 and 2.18 µg cm^−2^, respectively (Table 1).

In addition, we evaluated the possible role of fumigation toxicity of *O. syriacum* essential oil on *C. quinquefasciatus* adults over time. In fumigating assays, the LC_50_ of the essential oil was 12.1 µL L^−1^ after 1 h of exposure, strongly decreasing to 1.3 µL L^−1^ after 24 h of exposure. LC_90_ values were 28.8 and 2.2 µL L^−1^, respectively (Table 2). Following a similar trend, the fumigation LC_50_ of carvacrol was 8.2 µL L^−1^ after 1 h of exposure, strongly decreasing to 0.8 µL L^−1^ after 24 h of exposure. Moreover, LC_90_ values were 16.3 and 1.5 µL L^−1^, respectively (Table 3). Lastly, the lethal time (LT) values were calculated testing four concentrations of *O. syriacum* essential oil, ranging from 2.5 to 20 µL L^−1^; 66 min was the minimum LT_50_ value, obtained testing 20 µL L^−1^, while the LT_90_ was 103 min. Lower concentrations of this essential oil led to higher LT_50(90)_ values, namely 117 and 191 min testing 10 µL L^−1^, 201 and 408 min testing 5 µL L^−1^, and 426 and 789 min testing 2.5 µL L^−1^ (Table 3).

### 2.4. Inhibition of Acetylcholinesterase

**Figure 2:**
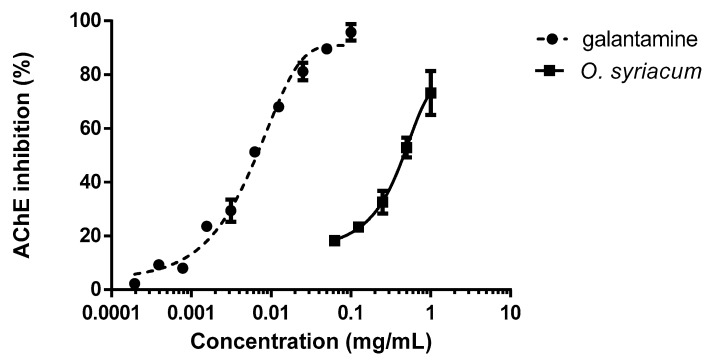
Inhibition of the acetylcholinesterase (AChE) by the *Origanum syriacum* essential oil over the positive control galantamine.

Finally, with the aim of identifying a possible mechanism of action, the inhibition of the AChE enzyme was evaluated. *O. syriacum* essential oil was able to inhibit the enzyme at doses that were considered larvicidal (Figure 2). Galantamine was tested as the positive control. Significant differences in AChE inhibition were observed according to the treatment concentration, testing both the oil (*F_4,10_* = 21.955; *p <* 0.001) and galantamine (*F_9,10_* = 120.292; *p <* 0.001) (Figure 2). The IC_50_ (half maximal inhibitory concentration) values were 0.461 and 0.007 mg mL^−1^, respectively.

In detail, concerning the experiments conducted testing with the *O. syriacum* essential oil, results pointed out that the inhibition of AChE enzyme reached values of 70% at concentrations of 1 mg mL^−1^. However, low concentrations of the essential oil that were larvicidal induced only 25% of AChE enzyme inhibition, which may reveal that other mechanisms can be involved at lower doses (Figure 2).

## 3. Discussion

The Lebanese accession of *O. syriacum* investigated here belonged to the carvacrol chemotype. It is worth noting that, based on these results, the carvacrol chemotype is not restricted only to cultivated plants as reported by Zein et al. [50]. In our study, the essential oil from *O. syriacum* was reported as an effective antiparasitic agent in the fight against anisakiasis, as well as a good larvicidal and adulticidal product to manage mosquito populations. The larvicidal activity of certain essential oils on *A. simplex* has been established [26,27,28,29,30,31,62]. Though other *Origanum* species have shown antiparasitic activity on *A. simplex*, *O. syriacum* seems to be the most promising, as it induces a higher mortality rate of the larvae at lower concentration. For example, a former study showed that *Origanum compactum* Benth had a LC_50_ value of 0.429 mg mL^−1^ at 24 h [31], which is more than four-fold lower (i.e., 0.087 mg mL^−1^) for *O*. *syriacum*. In another study a maximum *A. simplex* mortality rate as high as 53% was achieved testing *Origanum vulgare* essential oil [29], which reaches up to 100% for *O. syriacum* when tested at 0.125 mg mL^−1^. All these data suggest that *O. syriacum* essential oil is highly effective and more potent than essential oils from other *Origanum* species, with carvacrol being one of the most important compounds responsible for the larvicidal effects. In addition, the results of our assays pointed out that the *O. syriacum* essential oil treatment fully neutralizes the capacity of *A. simplex* larvae to penetrate agar, potentially inhibiting host muscle penetration and reducing the pathogenic capacity of the larvae [63].

The main constituents in the essential oil were the monoterpenes carvacrol, γ-terpinene, *p*-cymene, and thymol; some of these compounds, in particular, carvacrol and thymol, have recently shown larvicidal effects and acetylcholinesterase inhibitory activity as a potential mechanism of action [31]. However, the leaf essential oil of *O*. *syriacum* studied here showed significantly lower LC_50_ values (i.e., 0.08 and 0.067 mg mL^−1^ after 24 h and 48 h, respectively) on *A*. *simplex* L3, if compared with those achieved by carvacrol (LC_50_ = 0.176 and 0.178 mg mL^−1^, after 24 and 48 h, respectively) [31], outlining the potential synergistic effects due to the presence of minor constituents of the Lebanese oregano oil, a topic which surely deserves further research [62,64].

Furthermore, carvacrol and thymol are commonly found in *Origanum* species, being responsible for the antimicrobial activities of these plants [65,66]. In addition, carvacrol is more effective than its isomer thymol as a larvicidal agent and AChE inhibitor [31,67,68]. The increasing popularity of eating raw-undercooked fish together with certain fishing and processing procedures favoring the parasite cycle (e.g., fish evisceration at sea) contributes to a higher prevalence of anisakiasis. Currently, these types of gastrointestinal parasitic diseases are not pharmacologically treated, but considering results presented herein, *Origanum* essential oils, and particularly *O. syriacum*, might be industrially exploited with the aim of treating or preventing anisakiasis or as a fish food additive to avoid larvae propagation after evisceration.

Concerning the insecticidal activity of essential oils, mosquito larvicides with an LC_50_ lower than 100 ppm could be considered as promising [69]. In addition, this perspective enhances the possibility of growing such plants in monocultures where, through the application of appropriate cultivation technologies, sufficient biomass can be produced to extract essential oils [70]. In this framework, good examples of aromatic plants with interesting potential include *Foeniculum vulgare* Mill., *Coriandrum sativum* L., *Mentha longifolia* (L.) L., *Ocimum basilicum* L., *Pimpinella anisum* L., *Thymus* spp., and *Eucalyptus* spp. [69,71,72].

In addition, the adulticidal activity of this essential oil appeared to be due to both contact and fumigation activity, as shown in Table 1 and Table 2. The results of both types of tests show the prospective use of this essential oil as an active insecticidal or fumigant substance suitable for the elimination of adult mosquitoes in closed rooms. Based on the results of tarsal tests and estimated LC_90_ of 46.9 μg cm^−2^, it could be estimated that an effective concentration of about 0.5% can be used for contact spraying against mosquito adults. However, further semi-field and field tests are required to verify the effectiveness of our estimated concentration. Similarly, testing the capabilities of encapsulation technology and synergic relationship with other essential oils will help to increase the efficiency and prolong the duration of efficacy of potential botanical insecticides [73,74].

The good bioactivity of this essential oil can be ascribed to the major compound, carvacrol (82.6%), as showed by its low LC_50(90)_ values estimated here against *C. quinquefasciatus* larvae (29.5 mg L^−1^) and adults (tarsal test: 25.5 µg cm^−2^; fumigation test: 0.8 µL L^−1^ after 24 h of exposure), respectively. However, possible interactions with other minor components (*p*-cymene, γ-terpinene, and thymol) might be possible. Carvacrol is a monoterpene phenol considered as a typical marker of oregano. It is widely recognized as a potent antimicrobial and antioxidant agent and therefore used as a food preservative [66]. Together with its isomer thymol, carvacrol is classified as a Generally Recognized as Safe (GRAS) compound by the US Food and Drug Administration (FDA) so that its toxicity on mammals can be regarded as relatively low [75,76]. Indeed, its LD_50_ in rats, after gavage administration, is 810 mg/kg body weight [77]. Furthermore, carvacrol showed negligible effects on beneficial organisms such as mealworm beetles, honeybees, shellfish, and the mosquito fish *Gambusia affinis* Baird and Girard [78,79,80]. In our study, carvacrol proved to be highly effective against L3 larvae of *A. simplex* showing LD_50_ values of 0.176 mg mL^−1^ at 24 h and 0.178 mg mL^−1^ at 48 h. Furthermore, it inhibited the AChE enzyme as a possible target for its mode of action [31]. In this respect, the interaction with the GABA_A_ and octopamine receptors may also be responsible for its toxicity on parasites and pests [81,82]. In detail, concerning mosquitoes, carvacrol exhibited high toxicity against larvae of different species, including *C. quinquefasciatus*, *Culex tritaeniorhynchus* Giles, *C. pipiens*, *Anopheles stephensi* Liston, and *Anopheles subpictus* (Grassi) with LC_50_ values of 26.1, 28.0, 37.6, 21.2, and 24.1 ppm, respectively [52,83].

## 4. Materials and Methods

### 4.1. Plant Material

Leaves of *O. syriacum* were collected from plants naturally growing in a mountain named Awaida, close to the village of Tayibe (33°16′35′′N; 35°31′14′′E, 800 m above sea level), Marjeyoun district, South Lebanon, in May 2017. Taxonomic identification of the collected plants was performed by F. Bartolucci according to dichotomous keys and descriptions reported in Ietswaart [32] and Mouterde [84]. According to the characters observed and measured (i.e., stems and leaves tomentose, leaves acute with raised veins on the abaxial leaf surface, calyx c. 2 mm long) the collected plants belong to *O. syriacum*. Voucher specimens of the sampled populations are kept in the Floristic Research Centre of the Apennines (APP, acronym follows Thiers 2018) under the voucher codex APP No. 59012.

### 4.2. Isolation and Analysis of O. syriacum Essential Oil

Air-dried leaves (420 g) of *O. syriacum* were manually reduced into small pieces and then inserted into a 10 L flask filled with 6 L of distilled water and subjected to hydrodistillation using a Clevenger-type apparatus for 3 h. This process yielded 4.3% (w/w, n = 2, on a dry matter basis) of an orange essential oil. The essential oil was chemically characterized by GC-MS according to Benelli et al. [85].

### 4.3. Activities of O. syriacum Essential Oil against A. simplex

#### 4.3.1. Isolation of *A. simplex* Larvae

*A. simplex* L3 larvae were isolated from the intermediary host blue whiting *Micromesistius poutassou* (Risso) acquired from the fishmonger located at Villanueva de Gállego (Zaragoza). Larvae were washed with saline sterile solution of 0.9% NaCl (SS) and identified through light microscopy according to morphological features [86]. Only intact *A. simplex* s.l. L3 with length >2.0 cm were used.

#### 4.3.2. Larvicidal Activity on *A. simplex*

Ten larvae were introduced in each well of polystyrene six-well plates with a final volume of 2 mL containing different concentrations of the test solution as well as control wells without treatments [27]. *O. syriacum* essential oil was tested on *A. simplex* in the range of 0–1 mg mL^−1^. The parasites were incubated at 37°C in 5% CO_2_ for 24–48 h. *O. syriacum* essential oil-based treatments and the control were tested in triplicates on three different days. Larvae were examined at 24 and 48 h under a microscope and immobile L3 were considered dead. Levamisole was used as the positive control of dead *A. simplex* larvae.

#### 4.3.3. Penetration Assays

After calculating LC_50_ values and testing the larvicidal capacity, the penetration assay was run. This assay is performed to simulate and reproduce the capacity of the larvae to penetrate the host muscle by using a specific medium. Agar block plates were prepared in six-well plates with the aim of studying the penetration ability of infective larvae [31]. The agar solution was made with the following reagents: 1% agar in RPMI-1640 Medium solution (pH 4, Sigma, Ronkonkoma, NY, USA) with 20% Foetal Bovine Serum (Lonza, Salisbury, MD, USA). Four milliliters of the solution were poured into each well. Then, 100 μL of supernatant, RPMI-1640 (RPMI-1640, 20% FBS, 1% commercial pepsin, pH 4.0), was placed into each well. *A. simplex* L3 were incubated with previously estimated LC_50_ values of *O. syriacum* essential oil for 1 h. Larvae were washed with SS and ten worms were placed on each control or sample well. Every condition was tested in triplicates. Plates were placed at 37 °C in 5% CO_2_ and the number of L3 larvae that penetrated the solid agar block was counted after 1, 12, and 24 h of incubation.

### 4.4. Larvicidal Activity on C. quinquefasciatus

*C. quinquefasciatus* third instar larvae were reared at 25 ± 1 °C, 70% ± 3% R.H. and 16:8 h (L:D) as recently reported by Benelli et al. [85]. Then, larvicidal assays were done testing the *O. syriacum* essential oil in dimethyl sulfoxide (DMSO) following Benelli et al. [85]. The *O. syriacum* essential oil and its major constituent, carvacrol, were tested at concentrations of 10, 20, 30, 40, 50, 60, 80, and 100 mg L^−1^ to estimate the LC_50(90)_ values (four groups, each composed of 25 larvae, were tested per concentration). Distilled water + DMSO used to formulate the *O. syriacum* essential oil was the negative control. α-Cypermethrin (Vaztak^®^), a widely used commercial insecticide also effective on *Culex* mosquito larvae, among others, was the positive control (concentrations: 0.0005, 0.001, 0.002, 0.003, 0.004, and 0.005 mg L^−1^). In all controls, four groups, each composed of 25 larvae, were tested. Mortality was recorded after 24 h. The assays were placed in a growth chamber [16:9 (L:D), 25 ± 1 °C].

### 4.5. Tarsal Contact Test on C. quiquefasciatus Adults

Tarsal toxicity on mosquito adults was studied following the World Health Organization method [86] with minor changes by Pavela (2014) [72]. *O. syriacum* essential oil or its major constituent, carvacrol, was formulated in 2 mL of acetone plus silicon oil (3.6 mg cm^−2^) and then provided on Whatman no. 1 filter paper (12 × 15 cm), testing seven doses (i.e., 5.0, 10.0, 20.0, 30.0, 40,0, 50.0, and 60.0 μg cm^−2^, four groups, each composed of 20 insects, were tested per concentration). α-Cypermethrin (Vaztak^®^) was the positive control (concentrations: 0.5, 1.0, 1.2, 1.5, 2.0, 2.5, and 3.0 μg cm^−2^). Concerning negative controls, mosquitoes were exposed to filter paper pretreated with the same amount of acetone + silicon oil, without *O. syriacum* essential oil. In all controls, four groups, each composed by 20 insects, were tested. In all cases, filter paper was then dried at 22 °C for 24 h, and placed in test tubes [72]. Twenty non-blood-fed adult females (one to three days old) were then exposed to the treated paper for 60 min. Therefore, mosquitoes were stored in plastic cages (20 × 20 × 20 cm) and fed ad libitum with a sucrose solution. Mortality was determined after 24 h. The insects were placed in a growth chamber [16:9 (L:D), 25 ± 1 °C].

### 4.6. Fumigation Test on C. quiquefasciatus Adults

The adulticidal activity of *O. syriacum* essential oil and its main constituent, carvacrol, through fumigation was assessed relying to airtight fumigation assays, in agreement with the method by Pavela [72]. Twenty non-blood-fed females (two to six days old) were placed in 250 mL conical flasks. Then, we added five doses of *O. syriacum* essential oil or carvacrol (from 8 to 30 μL L^−1^ and from 0.5 to 5.0 μL·L^−1^ for 1 and 24 h exposition, respectively; for each concentration, four groups, each composed of 20 insects, were tested) in acetone, dropping 10 μL of the mixture onto filter paper (1 × 3 cm). Conical flasks were sealed as detailed by Pavela [72]. Control was treated under the same conditions with pure acetone (four groups, each composed of 20 insects, were tested). Mortality was noted after 1 or 24 h. The assays were placed in a growth chamber [16:9 (L:D), 25 ± 1 °C].

### 4.7. Lethal Time Assessment on C. quiquefasciatus Adults

The theoretic fumigation exposure time needed to achieve mortality of mosquito adults was determined in a series of experiments carried out using identical methods as above (Section 4.6. Fumigation test on *C. quiquefasciatus* adults) with the unique difference that mortality was recorded over time, i.e., every 5 min during the first 30 min, and then every 10 min from 30 to 200 min of the assay. The *O. syriacum* essential oil was formulated at 20.0, 10.0, 5.0, and 2.5 µL L^−1^ to assess its toxicity on female adults of *C. quinquefasciatus*. To estimate the lethal time (LT_50,90_) needed to achieve 50% or 90% mortality, seven time intervals were selected where mortality was noted from 10% to 95%. The assays were placed in a growth chamber [16:9 (L:D), 25 ± 1 °C]. Each experiment was replicated three times.

### 4.8. Inhibition of Acetylcholinesterase

AChE inhibition by the essential oil of *O. syriacum* was performed and quantified in 96 microplates using the Ellman method [87] with minor modifications. In this assay, each well contained 25 µL of 15 mM acetylthiocholine iodide in Millipore water, 125 µL of 3 mM DTNB in buffer C (50 mM Tris-HCl, pH 8, 0.1 M NaCl, 0.02 M MgCl_2_6H_2_O), 50 µL buffer B (50 mM Tris-HCl, pH 8, 0.1% bovine serum), and 25 µL of *O*. *syriacum* essential oil at different concentrations. The *O. syriacum* essential oil was diluted in DMSO and tested in triplicates over different days. Then, 25 µL 0.22 U/mL AChE was added and the absorbance was measured at 405 nm using a kinetic mode. Galantamine was tested as the positive control.

### 4.9. Statistical Analysis

In anthelminthic assays, all experiments were performed in triplicates over different weeks using new *A. simplex* larvae. LD_50_ (median lethal dose) for *A. simplex* larvicidal activity was calculated using nonlinear regression (GraphPad Prism 5). Data were subjected to analysis of variance, and mean comparison was performed by one-way ANOVA plus Scheffe’s multiple comparisons (*p* ≤ 0.05). Statistical analysis was performed using PASW Statistics 18. In mosquito larvicidal and adulticidal assays, when mortality in the control ranged from 1% to 20%, we corrected experimental mortality with Abbott’s formula [88]; if control mortality was >20%, experiments were repeated. LC_50(90)_ as well as LT_50(90)_ related parameters detailed in Table 1, Table 2 and Table 3 were estimated using probit analysis [89]. AChE inhibition data were transformed (arcsine √) and analyzed using ANOVA followed by Tukey’s HSD test. *p* = 0.05 was used as a threshold to separate means; the IC_50_ (half maximal inhibitory concentration) of galantamine and the tested essential oil were calculated as described above for *A. simplex* using nonlinear regression.

## 5. Conclusions

The findings of our study highlighted that the *O. syriacum* essential oil is highly effective against the filariasis vector *C. quinquefasciatus* and the parasite *A. simplex*. Notably, its bioactivity is related to the high content of carvacrol, a phenolic monoterpene. The possibility of developing effective, eco-friendly, and safe botanical insecticides with this essential oil is high. Indeed, scalability is assured by both wild and cultivated accessions of this species that occurs in several Middle East countries. Moreover, these prospects are enhanced by the fact that *O. syriacum* is currently grown as a commercial crop, and provided that a suitable growing technology is used, more than 4500 kg of dry mass can be obtained from one hectare, yielding about 180 kg of essential oil. Thus, the crop may provide an easily available and relatively inexpensive source of active substances for potential botanical-based drugs and insecticides, which can be further stabilized through nano- and microemulsions [90] and proposed for real-world control programs under the IVM framework. 

## Figures and Tables

**Table 1:** Efficacy of *Origanum syriacum* essential oil and its main constituent, carvacrol, against larvae and adults of *Culex quinquefasciatus*.

Target Insect	Unit	LC_50_	CI_95_	LC_90_	CI_95_	*χ^2^*
***O. syriacum* Essential Oil**						
*C. quinquefasciatus* third instar larvae	mg L^−1^	32.4	31.3–33.6	40.1	38.3–42.3	6.396 *ns*
*C. quinquefasciatus* adult females (tarsal toxicity test)	µg cm^−2^	28.1	25.9–30.3	46.9	42.1–54.1	4.698 *ns*
**Carvacrol**						
*C. quinquefasciatus* third instar larvae	mg L^−1^	29.5	28.3–31.8	39.2	36.7–42.9	5.214 *ns*
*C. quinquefasciatus* adult females (tarsal toxicity test)	µg cm^−2^	25.5	21.2–27.3	35.8	32.7–41.5	3.251 *ns*
**Positive Control, α-Cypermethrin**						
*C. quinquefasciatus* third instar larvae	mg L^−1^	0.0008	0.0006–0.0012	0.0025	0.0021–0.0032	5.235 *ns*
*C. quinquefasciatus* adults (tarsal toxicity test)	µg cm^−2^	1.22	0.95–1.38	2.18	2.01–2.26	3.245 *ns*

*ns* = not significant (*p* > 0.05).

**Table 2:** Fumigation toxicity of *Origanum syriacum* essential oil and its main constituent carvacrol against adults of *Culex quinquefasciatus*.

Treatment	LC_50_ (µL L^−1^)	CI_95_	LC_90_ (µL L^−1^)	CI_95_	*χ^2^*
***O. syriacum* Essential Oil**					
1 h of exposure	12.1	10.8–13.6	28.8	24.7–37.6	2.263 *ns*
24 h of exposure	1.3	1.3–1.5	2.2	1.9–2.6	1.159 *ns*
**Carvacrol**					
1 h of exposure	8.2	7.9–10.7	16.3	15.9–19.3	2.152 *ns*
24 h of exposure	0.8	0.7–1.1	1.5	1.3–1.8	2.313 *ns*

*ns* = not significant (*p* > 0.05).

**Table 3:** Lethal time values estimated testing the *Origanum syriacum* essential oil on *Culex quinquefasciatus* adults.

Parameter	LT_50_ (min)	CI_95_	LT_90_ (min)	CI_95_	*χ ^2^*
Lethal time (LT_50,90_) for 20 µL L^−1^	66	62–69	103	97–109	2.239 *ns*
Lethal time (LT_50,90_) for 10 µL L^−1^	117	111–124	191	173–218	3.324 *ns*
Lethal time (LT_50,90_) for 5 µL L^−1^	201	185–222	408	343–537	4.957 *ns*
Lethal time (LT_50,90_) for 2.5 µL L^−1^	426	415–438	789	768–826	3.362 *ns*

*ns* = not significant (*p* > 0.05).
